# Using a global diversity panel of *Cannabis sativa L.* to develop a near InfraRed-based chemometric application for cannabinoid quantification

**DOI:** 10.1038/s41598-023-29148-0

**Published:** 2023-02-08

**Authors:** Francine Gloerfelt-Tarp, Amitha K. Hewavitharana, Jos Mieog, William M. Palmer, Felicity Fraser, Omid Ansari, Tobias Kretzschmar

**Affiliations:** 1 Southern Cross University, Lismore, NSW 2480 Australia; 2 Research Division, Rapid Phenotyping (Hone), Newcastle, NSW 2300 Australia; 3 Ecofibre Ltd, Virginia, QLD 4014 Australia; 4 Hemp GenTech, Fig Tree Pocket, QLD 4069 Australia

**Keywords:** Natural products, Infrared spectroscopy

## Abstract

*C. sativa* has gained renewed interest as a cash crop for food, fibre and medicinal markets. Irrespective of the final product, rigorous quantitative testing for cannabinoids, the regulated biologically active constituents of *C. sativa*, is a legal prerequisite across the supply chains. Currently, the medicinal cannabis and industrial hemp industries depend on costly chromatographic analysis for cannabinoid quantification, limiting production, research and development. Combined with chemometrics, Near-InfraRed spectroscopy (NIRS) has potential as a rapid, accurate and economical alternative method for cannabinoid analysis. Using chromatographic data on 12 therapeutically relevant cannabinoids together with spectral output from a diffuse reflectance NIRS device, predictive chemometric models were built for major and minor cannabinoids using dried, homogenised *C. sativa* inflorescences from a diverse panel of 84 accessions. Coefficients of determination (r^2^) of the validation models for 10 of the 12 cannabinoids ranged from 0.8 to 0.95, with models for major cannabinoids showing best performance. NIRS was able to discriminate between neutral and acidic forms of cannabinoids as well as between C_3_-alkyl and C_5_-alkyl cannabinoids. The results show that NIRS, when used in conjunction with chemometrics, is a promising method to quantify cannabinoids in raw materials with good predictive results.

## Introduction

*Cannabis sativa L.* (*C. sativa*), an annual herbaceous species of the Cannabaceae^1,2^, is a monotypic genus containing several subspecies (e.g. *C. sativa ssp sativa* and *C. sativa ssp indica*)^4^*.* Apart from taxonomic discrimination at sub-species level, *C. sativa* remains clearly distinguished legislatively, based on chemotypes determined by Δ9-tetrahydrocannabinol (Δ9-THC) concentrations^5,6^. Generally, *C. sativa* with Δ9-THC above 0.3% (w/w) are considered drug-type cannabis and those below 0.3% (w/w) are considered industrial hemp^7,8^. Legal definitions, however, vary between jurisdictions and can be less or more stringent in respect to Δ9-THC thresholds.

*C. sativa* has a long history of cultivation for food, fibre, recreation and medicine^9,10^. A near-global distribution, coupled with a propensity for outcrossing of artificial populations with natural populations and stable feral populations, created extensive variation in its morphology, phenology and chemical composition^11^. *C. sativa* is chemically complex, containing several classes of valuable secondary metabolites, including cannabinoids, terpenoids, and phenolic compounds^13^. While terpenoids and phenolics are abundantly found across the plant kingdom, cannabinoids, a group of terpenophenolic secondary metabolites are unique to *C. sativa*^14–16^. While over 150 cannabinoids have been identified, only a fraction are currently monitored routinely, primarily for the medicinal Cannabis industry^18^.

Cannabinoids are synthesised in their carboxylic acid (COOH) forms and decarboxylated to neutral bioactive cannabinoids via heat or light^19,20^. Δ9-THCA and cannabidiolic acid (CBDA), classified as C_5_-alkyl cannabinoids, are the most abundant and best characterised. They share cannabigerolic acid (CBGA) as a common precursor, which is catalysed into Δ9-THCA via THCA synthase, and/or catalysed to CBDA via CBDA synthase. CBGA, in turn, is formed via olivetolic acid cyclase from olivetolic acid and geranyl pyrophosphate. Other less abundant therapeutically beneficial cannabinoids include C_5_-alkyl cannabichromenic acid (CBCA) and cannabinolic acid (CBNA)^21–23^. It was recently demonstrated that the bioactivity of cannabinoids on the human endocannabinoid system (ECS) is modulated by differences in alkyl chain length^24^, affecting the binding affinity of cannabinoids to respective ECS receptors. This generated interest in the therapeutic potential of C_3_-alkyl cannabinoids, including tetrahydrocannabivarinic acid (THCVA) and cannabidivarinic acid (CBDVA)^24^, which were consequently included in the scope of this study.

A renewed recognition of its therapeutic and nutraceutical values, coupled with reassessments of its risks, catalysed a global trend towards the deregulation of cannabis and hemp. Over the past decade, the medicinal and recreational cannabis industries have grown into multi-billion-dollar markets^25^ and research and development (R&D) into *C. sativa* is expanding^26,27^. R&D and industry have a high demand for compliance and quality assurance testing to monitor cannabinoid variability and develop processes that ensure consistent product quality^28^. Concomitantly, products derived from hemp fibre or seed have seen a renewed resurgence, marketed as healthy, sustainable and environmentally friendly^29^. Hemp industries primarily need cannabinoid testing to assure that Δ9-THC levels remain below the compliance thresholds of local jurisdictions.

Both industries largely rely on chromatographic methods such as High-Performance Liquid Chromatography coupled with ultra-violet spectrometry (HPLC–UV) or Mass Spectrometry (HPLC–MS), or Gas Chromatography (GC) in conjunction with Mass Spectrometry (GC–MS) or Flame Ionised Detection (GC-FID), for cannabinoid detection and quantification^30,31^. Despite being accurate and standard for certification purposes, these methods are time and cost-intensive. There is a growing demand for fast, robust, and inexpensive platforms for large scale testing in both hemp and medical cannabis industries.

Alternative analytical techniques such as Near-InfraRed Spectroscopy (NIRS) have gained popularity in secondary metabolite testing^32,33^. Advantages include non-destructive sample preparation, speed and low cost, while providing good predictions of secondary metabolite composition and content^34^. NIRS uses the absorption of electromagnetic radiation between 780 and 2500 nm to build a unique spectral fingerprint for each sample^35^. The interpretation of the spectral output depends on correlation data with a reference method such as HPLC to produce predictive models using chemometrics^36^. Importantly, the final models produced are highly influenced by the quality and quantity of the training set, data transformation, and algorithms used^37,38^.

A limiting factor for developing NIRS as an analytical tool for cannabis and hemp is access to large and diverse sample sets to sufficiently represent genetic and corresponding chemotypic diversity. The inclusion of additional minor C_3_-alkyl cannabinoids as targets and separation of carboxylic and neutral cannabinoids exacerbates these challenges. Most NIRS applications targeting cannabinoids *in planta* remain limited to semi-quantitative applications such as discriminating drug and fibre type chemovars^39,40^. For quantitative applications of NIRS, the emphasis has been on Δ9-THC determination^41,42^ or the determination of neutral cannabinoids^43^.

This research aimed to demonstrate the feasibility of NIRS combined with chemometrics to quantify a range of cannabinoids in diverse chemotypic backgrounds, with emphasis on the ability to distinguish between carboxylic and neutral forms of each cannabinoid and between C_5_ and C_3_- alkyl cannabinoids. Target cannabinoids were Δ9-THCA, Δ9-THC, CBDA, CBD, CBGA, CBG, THCVA, Tetrahydrocannabivarin (THCV), CBDVA, Cannabidivarin (CBDV), Cannabinol (CBN), and Cannabichromene (CBC) assessed in a global diversity set of *C. sativ*a.

## Results

### Chemotypic characterisation of the germplasm collection

**Figure 1:**
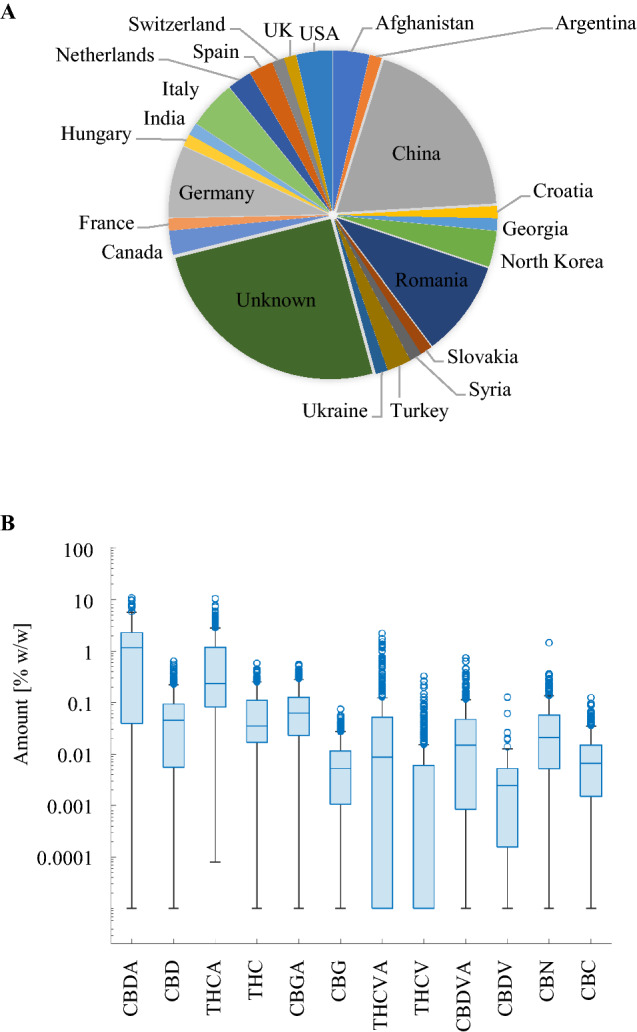
Geographic distribution and cannabinoid abundance of 84 accessions. (**A**) geographic distribution of the 84 accessions based on passport data. (**B**) Cannabinoid abundance of 12 cannabinoids across 249 samples, obtained from the HPLC reference analysis. Open circles represent outlier cannabinoid measurements above one standard deviation. The median value is represented by the line in the box, while the T bars are the lower and upper quartiles. A log-10 scale was applied on the y-axis. Cannabidiolic acid (CBDA), Cannabidiol (CBD), Δ9-Tetrahydrocannabinol acid (THCA), Δ9-Tetrahydrocannabinol (THC), Cannabigerolic Acid (CBGA), CBG (Cannabigerol), Tetrahydrocannabivarinic acid (THCVA), Tetrahydrocannabivarin (THCV), Cannabidivarinic acid (CBDVA), Cannabidivarin (CBDV), Cannabinol (CBN), Cannabichromene (CBN).

A total of 249 individual female plants, derived from 84 unique accessions (Supplementary Information Table S1), were subjected to HPLC-based cannabinoid quantification. The collection constituted a mix of cannabis (drug) and hemp type accessions. Using 0.3% (w/w) Δ9-THC as a threshold of distinction categorized 33 accessions as drug type and 51 as hemp. Collectively they originated from at least 22 countries (Fig. 1A), with a considerable proportion (27%) of unknown origin. The largest known proportion originated from China (15%), followed by Romania (10%) and Germany (9%). Based on a prior study, a substantial subset of the collection was explicitly selected for the presence of C_3_ alkyl cannabinoids^44^.

Except for THCV present in only 47% of samples, all other target cannabinoids were detected in over 60% of samples (Supplementary Information Table S2). The C_5_-alkyl side chain cannabinoids Δ9-THC and Δ9-THCA were detected in every sample, CBDA and CBGA were reported in 98% of samples, CBD in 96% of samples, followed by CBN in 95%, CBG in 88% and CBC in 87% of all samples tested. The C_3_-alkyl side chain cannabinoid THCVA was detected in 77% of the samples, CBDVA in 75%, CBDV in 69% and THCV in 47% of the samples analysed.

Where measured, cannabinoids displayed a higher range in their acidic form than their decarboxylated form (Fig. 1B, Supplementary Information Table S2). The largest range in concentration was found for Δ9-THCA with a minimum of 0.001% (w/w) and a maximum of 10.51% (w/w), whereas CBDA ranged from 0 to 9.76% (w/w). These ranges were approximately five times larger than the next biggest range for Δ9-THC 0.001–1.85% (w/w) and 0–1.75% (w/w) for THCVA. Among the neutral cannabinoids only, Δ9-THC (0.001–1.85% (w/w)) and CBD (0–0.52% (w/w)) had a median concentration between 0.01% (w/w) and 0.1% (w/w). CBG (0–0.08% (w/w)) CBC (0–0.12% (w/w)), CBDV (0–0.13% (w/w)) and THCV (0–0.26% (w/w)) had the lowest ranges with median concentrations of less than 0.01% (w/w).

The distribution of cannabinoids was highly skewed towards the lower concentration ranges. For example, Δ9-THCA with a maximum of 10.51% (w/w) only displayed a mean of 1.01% (w/w). This trend was observed for all 12 target cannabinoids, where mean values were closer to the lowest concentration than the highest concentration. Our collections did not contain any modern drug cultivars, which typically report 15–20% Δ9-THCA.

**Figure 2:**
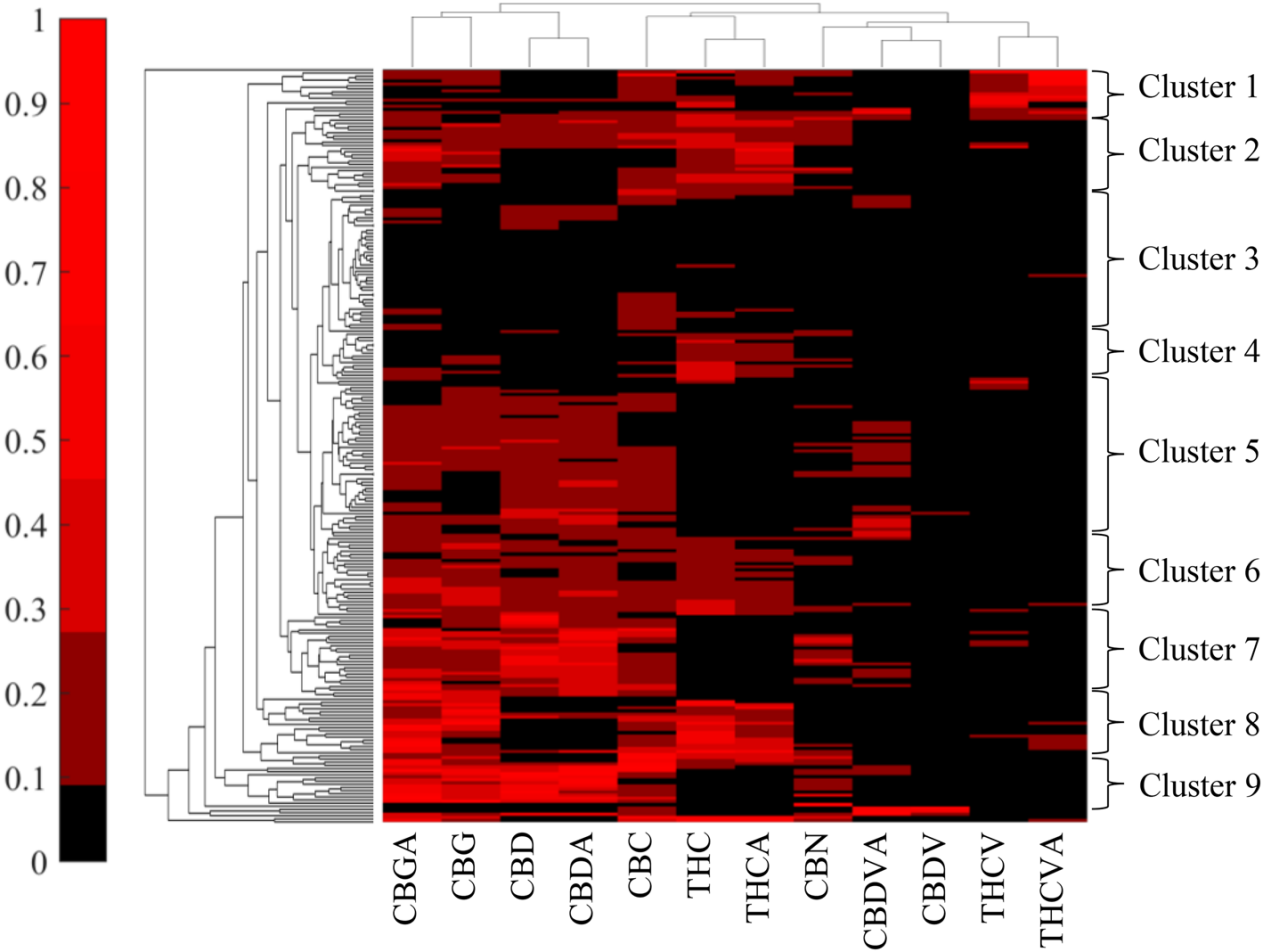
Hierarchical clustering of germplasm collection, normalised. The vertical dendrogram defines the relationship between samples/accessions, while the horizontal dendrogram defines the relationship between individual cannabinoids across each sample. Cannabidiolic acid (CBDA), Cannabidiol (CBD), Δ9-Tetrahydrocannabinol acid (THCA), Δ9-Tetrahydrocannabinol (THC), Cannabigerolic Acid (CBGA), Cannabigerol (CBG), Tetrahydrocannabivarinic acid (THCVA), Tetrahydrocannabivarin (THCV), Cannabidivarinic acid (CBDVA), Cannabidivarin (CBDV), Cannabinol (CBN), Cannabichromene (CBC).

Hierarchical clustering (Fig. 2) revealed relationships among samples based on their cannabinoid contents (vertical axis) and between individual cannabinoids (horizontal axis). There was good clustering between neutral and acidic forms for all cannabinoids (e.g. THCVA and THCV, CBDA and CBD) along the horizontal axis. CBDA and CBD were further clustered with CBGA and CBG and Δ9-THCA and Δ9-THC clustered with CBC, while all four C_3_ alkyl side chain cannabinoids (THCVA and THCV, CBDVA and CBDV) clustered together with CBN.

Vertically, nine clusters were apparent, based on relative cannabinoid abundances (Fig. 2, Supplementary Information Table S3). Cluster 1 contained samples with intermediate to high THCVA and/or intermediate Δ9- THC(A) content, with most accessions from China. Cluster 2 grouped intermediate to high Δ9-THCA and/or CBGA content samples. Cluster 3, the second-largest cluster, contained successions with low cannabinoid abundance for all 12 cannabinoids, consistent with fibre types and further supported by known variety names for several accessions in this group. Cluster 4 included samples with intermediate to high Δ9-THC and Δ9-THCA contents while being low in most other cannabinoids, which is indicative of drug types. Samples within cluster 5, the largest cluster, exhibited intermediate CBDA and a range of CBDVA and CBGA concentrations while being consistently low in Δ9-THCA and THCVA. Geographically, cluster 5 comprised mostly accessions from Eastern Europe. Cluster 6 contained accessions of mostly intermediate concentrations of C_5_ cannabinoids while being very low or devoid of C_3_ cannabinoids. Cluster 7 grouped accessions with relatively high CBDA and lower Δ9-THCA concentrations, with a range in CBGA, CBDVA and CBN. Cluster 8 comprised accessions with high Δ9-THCA, mostly high CBGA and a range of CBC and generally with intermediate THCVA concentrations. Cluster 9 samples were high in CBDA-CBD and CBGA-CBG, while intermediate in CBN in CBC. Adjacent to cluster 9 were two accessions of high CBDVA-CBDV content and the accession of the highest Δ9-THCA content. While individuals from the same accessions tended to cluster together, this was not always the case. For example, for accession SC1900022, eight individual samples were distributed across three clusters (3, 5 and 7; Supplementary Information Table S3).

### Spectral features

**Figure 3:**
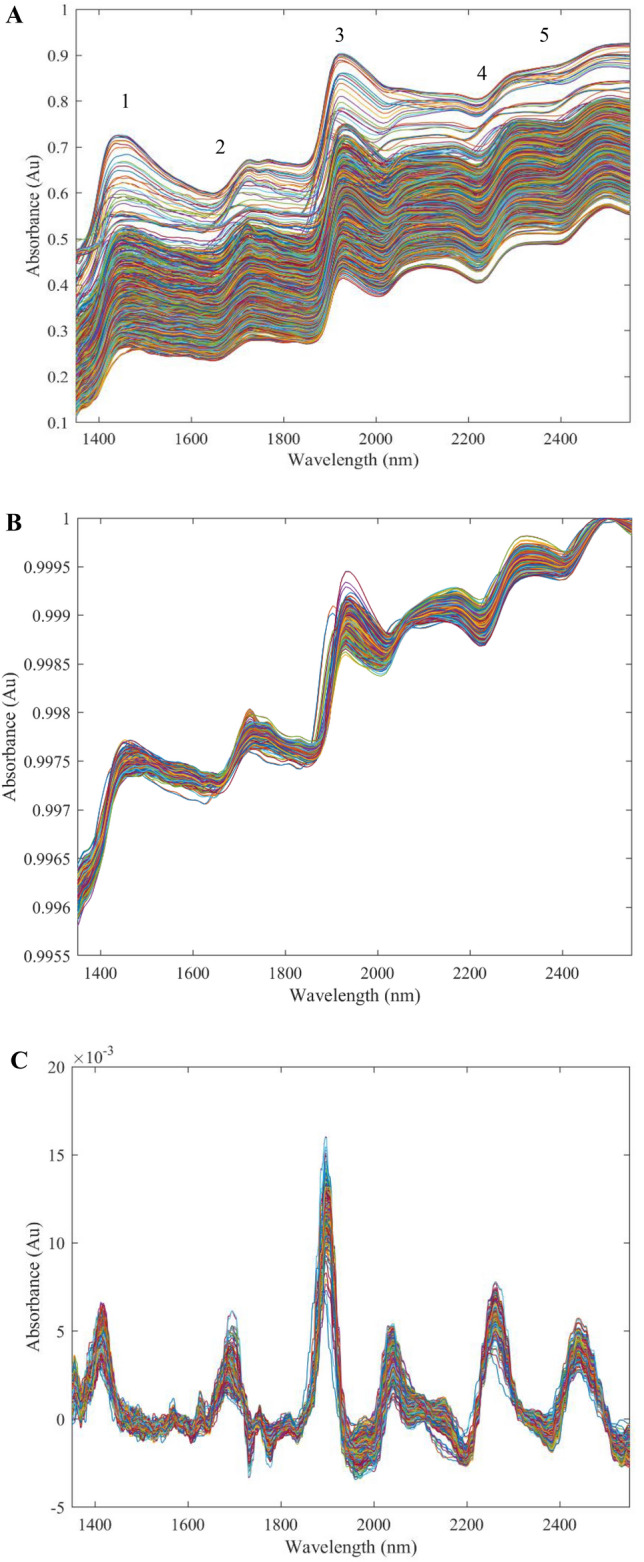
Pre and Post-processed spectra of the complete set of samples (n = 249) obtained using the NIR diffuse reflectance spectrometer in the wavelength region 1350–2500 nm. (**A**) The pre-processed (in absorbance) raw spectra for all 249 samples, including triplicate technical replicates. (**B**) The processed spectra for the models that were not derivatised. Includes models: CBN (19,097), CBC (19,096), CBGA (19,164), CBG (19,164), CBDV (17,199), THCV (19,161) and CBD (19,156). (**C**) Processed spectra that have been derivatised under first order derivatisation for CBDA (19,137), Δ9-THC (19,158), Δ9-THCA (19,179) and THCVA (19,180). For this figure a derivative data transformation was undertaken. The y-axis is the absorbance of reflected molecules in the test samples, the x-axis is the measured wavelength (nm). All spectra values were obtained from the Hone Create Platform. All processed spectra were determined from the software algorithms.

The raw spectra of dried samples from all 249 individual female C. *sativa* plants obtained using the NIR region between 1350 and 2500 nm are shown in Fig. 3A. The most common data transformations used (Supplementary Information Table S4) were area normalisation (seven out of the 12 cannabinoids) and 1st order derivatisation (4 out of the 12 cannabinoids). Smoothing and baseline shifts were applied to 3 out of the 12 cannabinoids. Derivative data transformations (Fig. 3C) appeared most suited to cannabinoids with the largest concentration ranges. Other data transformations resulted in a processed spectrum similar to the raw spectra (Fig. 3B).

Typical absorption bands in the NIR region of plant material can be identified^43^. Five major absorption bands were evident (Fig. 3A, annotated with numbers 1–5), correlating with specific chemical compositions. Based on literature, two major absorptions bands at around 1900–2000 nm (band 3) and around 1400–1500 nm (band 1), were likely indicative of the presence of an OH group^45^. Band 3 was primarily due to the presence of water^6^, whereas band 1 was likely due to an OH group attached to an aromatic ring^45^. At around 2200 nm, band 4 was associated with a combination of stretching and bending vibrations of CH_2_ and CH_3_ groups found in the backbone and sidechains of proteins and lipids^43,46^. At around 2400 nm, band 5 was associated with CH stretching and ring deformations^45^. Absorption band 2 at around 1700 nm was likely due to aromatic hydrocarbons typically found in terpenoids due to the presence of CH stretching and combination bands of the first overtone^6,45^. As NIR combination bands are non-specific to compounds due to similarities in functional groups, unique visible spectral features for cannabinoids could not be identified.

### Model building and validation

All model training data were supplied to a series of four machine learning algorithms on the Hone Create platform. The selected machine learning algorithms that provided the best performing model are summarised in Supplementary Information Table S4 under model type. For this purpose, a stacked ensemble was selected for ten of the 12 cannabinoids, and a gradient boosting machine was selected for the remaining two cannabinoids. All processing algorithms used can be found in Supplementary Information Table S5. For most models, except for CBDV, the three technical replicates were left unmerged.

Predictive chemometric models trained using HPLC reference values and corresponding NIRS spectra from 249 individual samples gave good to reasonably accurate estimations for 10 out of the 12 cannabinoids modelled (Table 1). Overall, CV r^2^ values were very high (0.98–1) with one exception (CBDV) which was considered a failed model (r^2^ = 0.124). Disregarding CBDV, CV root mean standard errors (RMSE) as percent of range was highest for THCVA at 4.05% and THCV at 3.62%. All other CV RMSE % of the range were below 2.5%, with the lowest recorded for CBN at 0.46%.

**Table 1:** Summary statistics for the best chemometric evaluation models. Cross-validation (CV) at 75% of sample size, hold-out validation (HV) at 25% of sample size, number of samples (n) used to build or validate the model.

Cannabinoid	Model number	Validation	n	min % (w/w)	Max % (w/w)	mean % (w/w)	r^2^	RMSE	RMSE % of range	RMSE % of mean
CBD	19,156	CV	537	0.01	0.52	0.06	**0.99**	0.01	2.4	12.56
		HV	175	0.02	0.42		**0.87**	0.04	5.86	30.72
CBDA	19,137	CV	697	0.01	9.72	1.68	**1.00**	0.16	1.62	9.4
		HV	228	0.01	9.76		**0.90**	0.56	5.79	33.59
Δ9-THC	19,158	CV	778	0.01	1.85	0.14	**0.99**	0.04	1.99	26.93
		HV	253	0.01	1.85		**0.95**	0.06	3.32	44.98
Δ9-THCA	19,179	CV	778	0.001	10.51	0.94	**0.98**	0.18	1.73	19.33
		HV	253	0.01	5.09		**0.95**	0.4	3.79	42.45
THCV	19,161	CV	413	0.01	0.26	0.02	**1.00**	0.01	3.62	37.74
		HV	137	0.01	0.26		**0.79**	0.02	6.65	69.45
THCVA	19,180	CV	593	0.01	1.75	0.14	**1.00**	0.07	4.05	50.08
		HV	197	0.01	1.74		**0.73**	0.02	7.7	95.18
CBDV	17,199	CV	255	0.01	0.13	0.01	**0.124**	0.01	6.38	247.66
		HV	38	0.01	0.01		**0.03**	0.003	2.36	91.73
CBDVA	19,162	CV	595	0.01	0.74	0.05	**1.00**	0.02	2.39	37.29
		HV	194	0.01	0.45		**0.50**	0.04	6.05	94.5
CBG	19,164	CV	296	0.01	0.08	0.02	**0.99**	0.001	1.85	5.27
		HV	98	0.01	0.06		**0.83**	0.006	7.54	21.54
CBGA	19,178	CV	689	0.01	0.74	0.11	**1.00**	0.02	2.48	16.61
		HV	232	0.01	0.74		**0.87**	0.04	5.9	39.52
CBC	19,096	CV	305	0.01	0.12	0.02	**1.00**	0.001	1.88	8.47
		HV	100	0.01	0.10		**0.87**	0.004	4.12	18.54
CBN	19,097	CV	513	0.01	0.76	0.07	**1.00**	0.01	0.46	5.33
		HV	171	0.01	0.29		**0.74**	0.03	4.61	52.82

“CV” is cross validation, “HV” is hold out validation, “n” is sample number, “min” is the minimum cannabinoid concentration, and “max” is the maximum cannabinoid concentration in percent of cannabinoid weight by sample dry weight (% (w/w)), “r^2^” is the regression coefficient, “RMSE” is the root mean square error. Significant values are in bold.

Statistical metrics for hold-out (HV) validation generally did not closely resemble the CV results (Table 1). Disregarding CBDV, the lowest HV r^2^ value was 0.50 for CBDVA, which was also considered a failed model as a result. For the remaining 10 cannabinoids, the average HV r^2^ was 0.85 with an RMSE as a percent of the range of 5.53%, which was 0.15 lower (r^2^) and 3.32% higher (RMSE % of range) than for the CV. Based on HV, the best performing model was Δ9-THC (19,158) with an r^2^ of 0.95 and an RMSE as a percent of range of 3.32%, followed by Δ9-THCA (19,179) with an r^2^ of 0.95 and 3.79% RMSE as a percent of range and CBDA (19,137) with an r^2^ of 0.90 and 5.79% RMSE of range. Models for CBD (19,156), CBGA (19,178), CBC (19,096) and CBG (19,164) performed moderately well with an r^2^ above 0.80 and RMSE between 4–6% of range. HV r^2^ above 0.70 were obtained for CBN (19,096), THCV (19,161) and THCVA (19,180), at an RMSE between 6 and 7%.

**Figure 4:**
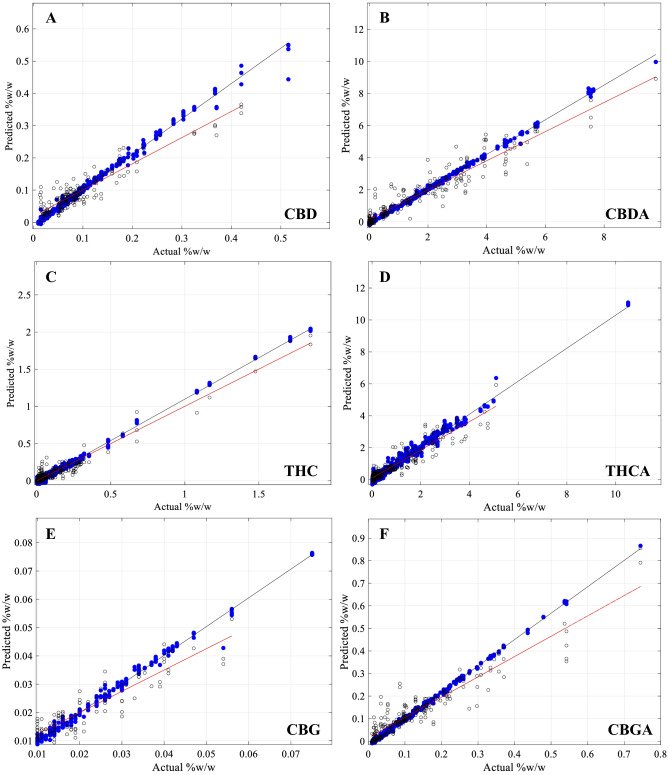

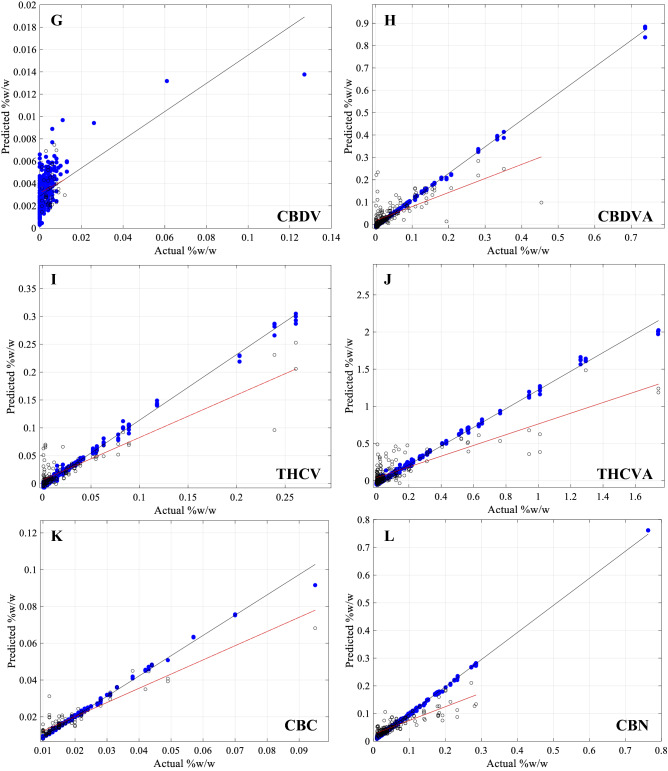
Cross-validation and Hold-out validation results for all 12 target cannabinoids. Actual values were obtained via HPLC reference method, predicted values were obtained from scans using the HL-EVT9-Neo NIR spectrometer, modelled using the Hone Create chemometric platform. Root mean square error and r^2^ values are given in Table 1. Cross-validation (CV), blue closed circles and black trend-line and Hold-out validation (HV), open circles and red-trend line results for the 12 target Cannabinoids: (**A**) Cannabidiol (CBD) (**B**) Cannabidiolic acid (CBDA), (**C**) Δ9-tetrahydrocannabinol (Δ9-THC), (**D**) Δ9-tetrahydrocannabinolic acid (Δ9-THCA), (**E**) Cannabigerol (CBG), (**F**) Cannabigerolic acid (CBGA), (**G**) Cannabidivarin (CBDV), (**H**) Cannabidivarinic acid (CBDVA), (**I**) Tetrahydrocannabivarin (THCV), (**J**) Tetrahydrocannabivarinic acid (THCVA), (**K**) Cannabichromene (CBC), (**L**) Cannabinol (CBN).

Graphical representation of correlation for both the CV (solid blue circles) and HV (open black circles) (Fig. 4) allowed for a direct comparison between CV and HV data and performance. Cannabinoid concentration ranges for Δ9-THCA, CBG, CBDV, CBDVA and CBN were smaller for the HV sets than the CV set. In all cases, the linear regression slope for the HV (Fig. 4; red-line) displayed a reduced slope compared to the slope of the CV (Fig. 4; black-line), indicative of under-predicting actual values at higher abundances. CBDV showed a lack of range and strong skewness towards zero. Scattered overprediction at very low (less than 0.01% (w/w)) concentrations for CBDV, CBDVA, THCV and THCVA resulted in a smearing of data points along the y-axis (Fig. 4). Most samples appeared to have reasonably accurate predictions at low cannabinoid concentration levels, where training data points were most abundant. For most cannabinoids, there was a sufficient distribution between the minimum and maximum values, although a prominent gap in data points was observed for CBN and to lesser extend for CBDVA and THCA.

Residual plots for all target cannabinoids (Supplementary information Fig. S1) displayed random scattering that did not suggest any heteroscedasticity. Variance was consistently higher for HV than for CV.

## Discussion

The Cannabis diversity panel of 84 accessions (Supplementary Information Table S1), representing at least 22 countries (Fig. 1A), constituted a good genetic resource for model training for several reasons. Firstly, all 12 target cannabinoids were represented at ranges typically found in *C. sativa* (Fig. 1B)^13,47^, including good representative ranges for the C_3_-alkyl cannabinoids THCVA and CBDVA. Cannabis samples with exceptionally high concentrations of Δ9-THCA and CBDA as found in modern medicinal cultivars (over 10% (w/w)), however, were underrepresented in this sample set, with maximum concentrations reaching 9–10% (w/w)^8,48^.

Importantly, a mean below 1% (w/w) for Δ9-THCA placed a large fraction of the samples in the range critical to determine hemp vs. drug-type cannabis, which typically lies between 0.3% and 1% (w/w) for Δ9-THC, depending on the jurisdiction^49^. When Δ9-THC was combined with Δ9-THCA using the formula $\Delta 9-THC+(\Delta 9-THCA*0.877)$, 48% of the samples were below the 0.3% (w/w) threshold and 65% of samples were below the 1% (w/w) threshold for total Δ9-THC, which is the legal threshold for industrial hemp in NSW Australia and used here to identify industrial hemp types for the germplasm collection (see Tables S1 and S2). In our sample set, cannabinoids with the largest % (w/w) concentrations and ranges were found in their acidic form compared to their neutral counterpart. Since our extraction and analysis protocols did not induce thermogenic decarboxylation, these numbers reflect the natural occurrence of cannabinoids *in planta*^15,19^. Models to detect the acidic counterparts of CBN and CBC could not be developed due to the absence of CBNA and CBCA in the reference method.

The diverse set of samples representing at least nine different combinations of cannabinoid composition (Fig. 2) ensured that models for individual cannabinoids were built across a range of different chemotypic backgrounds. On the vertical axis, the largest cluster was cluster 3, followed by cluster 5 (Fig. 2). Together, they made up 40% of all samples, with some of the lowest Δ9-THC and Δ9-THCA content in the germplasm set, representing the industrial hemp fibre type classification. Having overall low levels of cannabinoids was beneficial to validate NIRS models for lower concentrated cannabinoids, as it supports the application of NIRS for compliancy purposes of industrial hemp, for legal purposes to discriminate between Hemp and Cannabis and for screening purposes to detect minor C_3_-alkyl cannabinoids of medicinal interest. Cluster 1 grouped most accessions containing THCVA, which were largely of Chinese origin, confirming reports that this rare cannabinoid was typically found in Central Asian and Southern African accessions^23,50^. Cluster 4 contained intermediate Δ9-THC concentrations with low overall cannabinoid concentrations, suggesting a strong presence of THCA synthase in these chemotypes, likely intended for recreational use. In cluster 6 levels of CBDA and Δ9-THCA displayed similar intermediate concentrations, possibly due to recent breeding strategies for high CBD and Δ9-THC concentrations through the addition of hemp-type CBDA synthase gene clusters into drug type cannabis^56^.

Horizontally, C_3_-alkyl cannabinoids such as CBDV(A) and THCV(A) clustered together (Fig. 2), likely reflecting different metabolic processes pathways in cannabinoid biosynthesis. THCVA and CBDVA showed inverse relationship much like observed between CBDA and Δ9-THCA, as they equally rely on THCA synthase or CBDA synthase activity, respectively.

HV validation statistics were less favourable than for CV. Relatively higher RMSE and lower r^2^ (Table 1) in HV for most models (Table 1) suggested a degree of model over-fitting for target cannabinoids. This was supported by the observations that residual variance for HV was consistently higher than for CV (Supplementary information Fig. S1). Absence of any discernible patterns or trends in the residual plots for CV and HV data (Supplementary information Fig. S1), however, was indicative of good overall model performance. C_5_-alkyl cannabinoids performed better than C_3_-alkyl cannabinoids with key cannabinoids CBDA, CBD, Δ9-THCA and Δ9-THC showing the lowest RMSE % and the highest r^2^ values for HV. While it was reasonable to assume that models for CBDVA and CBDV failed due to low concentrations and lack of range of training data, other minor cannabinoids with smaller ranges and lower average concentrations, such as CBC and CBG, produced models of relatively good predictive power (r^2^ above 0.7), but had some of the highest RMSE % of range values for HV with CBG at 7.54 RMSE % of range. General constraints in the prediction of C_3_-alkyl cannabinoids are also unlikely, since THCVA and THCV models predicted with relatively high confidence (r^2^ above 0.7) but also had some of the highest RMSE % of range values for their HV validation. It is possible that the models for CBDVA and CBDV failed because accessions containing relatively high concentrations of CBDVA and CBDV were consistently high in CBDA and CBD in absolute terms as well (Fig. 2: clusters 5 and 7). This could have potentially confounded the discrimination by NIRS for the structurally related C_3_ cannabinoids. On the other hand, accessions containing high THCVA and THCV found in cluster 1 were low in Δ9-THC and Δ9-THCA, thus not masking the similar C_3_-alkyl cannabinoids.

Spectroscopic techniques for cannabinoid analysis have been utilised both for quantitative and qualitative analysis, with popular analytical methods aimed at distinguishing between fibre and drug type cannabis^6,39,41^, Other spectroscopic techniques using NIR to quantify multiple cannabinoids remain limited in publication, with often a maximum of eight cannabinoids reported and use larger benchtop devices^43,57^. However, these publications reported promising r^2^ values typically above 0.8 with similar RMSE. In respect to using NIRS to quantify cannabinoids, a lot of applications remain limited in their access to diverse germplasm collections, which is why assembling a diverse germplasm across a range of cannabinoid and cannabinoid concentrations was a priority, making this NIRS application novel in that approach.

This study shows for a first time that NIRS has the specificity to detect small molecular differences such as the loss of a COOH group between acidic and neutral cannabinoids and a difference of two CH_2_ for all the C_5_-alkyl cannabinoids. The ability to quantify the extent of decarboxylation in a non-destructive manner by NIRS would be particularly beneficial in shelf-life studies and quality control analysis, tracking potential non-enzymatic conversions under different processing or storage conditions. The ability to discriminate between the relative abundance of cannabinoids of alkyl side chain lengths at scale provides a novel opportunity in variety improvement where large-scale screening is necessary to select recombinants of improved cannabinoid profiles. Finally, the sensitivity of the NIRS to predict low cannabinoid concentrations, mainly Δ9-THCA and THCVA, will allow for critical compliance testing.

A key factor to consider when using NIRS predictions is the impact of reference and training data quality and quantity on the model quality. Considerations for the training data include the relationship of target samples to the training set, sample type, processing and storage^41^. Accurate predictions can only be made within the calibration ranges defined by the training set. This particular sample set was limited to low concentration ranges for the majority of the target cannabinoids. Though beneficial for hemp industries, using these models to predict higher cannabinoid concentrations typically found in medicinal cannabis^8^ could either underpredict or overpredict the true cannabinoid concentration. Calibration ranges can be extended by adding samples previously out of range to the training set and generating new models.

As shown in Fig. 4, HV samples tended to be underpredicted at higher concentrations. Cannabinoids at the higher concentration range from this sample set, however, were far fewer than lower concentrated cannabinoids (Fig. 4). Since the cannabinoids were not normally distributed (Fig. 1A), a fundamental assumption for machine learning algorithms and regression statistics^58,59^, it can have the tendency to skew the slope of the regression curve, which may be the reason why higher concentrated cannabinoids are underpredicting. Another limitation observed through this model building process was found for limit level cannabinoid concentrations. Smearing of values along the vertical axis (Fig. 4) was observed for the predicted values at the 0.01% (w/w) concentration level. This suggests a loss of sensitivity using the NIRS at these low limit concentrations.

Collectively, this study supports the use of NIRS as a supplementary or complementary form of analysis to conventional chromatographic analysis. Especially if its application takes the respective limitations for target cannabinoids into account, NIRS can provide estimations for decision making by being fast, non-destructive, scalable and cheap. Importantly, NIRS can reduce potential bottlenecks for analysis and screening for research and development applications. Examples of this include staged approaches which often necessitate large scale testing of trials, with aims to identify treatment effects during cultivation (e.g. light, nutrients, stresses), identify quantitative trait loci underlying chemotypes in mapping populations or reduce population sizes to numbers amenable for more detailed analysis in breeding^60,61^.

## Methods

*C. sativa* cultivation, sample, storage, processing of plant material and cannabinoid analysis were performed in strict adherence to Sectoins23(4)(b) and 41(b) of the NSW Drug Misuse and Trafficking Act 1985, held under the Authority granted to Prof. Bronwyn Barkla of Southern Cross University, issued by the New South Wales Ministry of Health, Australia. All 84 accessions represent cultivated materials, sourced from two germplasm collections, the Ecofibre Global Germplasm Collection (Ecofibre Ltd), labelled as ECO and the Hemp genebank collection of the Leibniz-Institute for Plant Genetics and Cultured Plant Research, labelled as IPK (Supplementary Information Table S1) . The private ECO collection was received and handled under a bilateral Material Transfer Agreement (MTA) between Southern Cross University and Ecofibre Ltd, while he IPK collection was received and handled under the FAO governed Standard Material Transfer Agreement (sMTA) (https://www.fao.org/plant-treaty/areas-of-work/the-multilateral-system/the-smta/en/). Both MTA were drafted in compliance with relevant institutional, national, and international guidelines and legislation. A total of 249 mature female inflorescences were grown and harvested and used for HPLC–UV and NIRS analysis in strict adherence to the respective MTAs.

Δ9-THCA, CBDA, Δ9-THC, and CBD reference standards were purchased from Novachem Pty Ltd (Heidelberg, Victoria, Australia), supplied in ampoules at a concentration of 1 mg/mL.

All solvents used for extraction and instrumental analysis were HPLC grade (Honeywell Research Chemicals, Seelze, Germany) and MilliQ water (18.2 MΩ cm^−1^, Merck Millipore Billerica, MA, USA).

### Growth parameters

Ten seeds from each accession were imbibed with 0.3% hydrogen peroxide solution on a paper towel and incubated in a SANYO growth cabinet (MLR-350, Osaka, Japan) under a 16 h light regime at 28 °C. Germinated seeds were transplanted at a depth of 1 cm into pots (75 mm × 100 mm) containing a soil mix of 1:1:1 vermiculite, peat moss, perlite and 100 g/100L of dolomite. Aqua Vega (CANNA^®^, Australasia) was used as liquid fertiliser. All plants were grown indoors at temperatures ranging between 26 and 32 °C and watered daily. Lighting was supplied by overhead LED lights (ViparSpectra R900 (900W) series) set to 12 h light 12 h dark cycles to stimulate flowering. Male progenies were removed to ensure unfertilised female inflorescences. For monoecious lines identifiable male flowers were plucked off on a daily basis. Samples were harvested when around 90% of the stigmas on the apical inflorescence had turned brown^62^.

### Post-harvest sample preparation

Samples were harvested by removing the main stems and larger fan leaves. Samples were dried for at least seven days at 15 °C under 15% relative humidity. Dried samples were homogenised using a 50 mL stainless steel grinding jar (Retsch GmbH, Germany) with a 20 mm stainless grinding ball (05.368.0062, Retsch GmbH, Germany) in a Mixer Mill MM 301 (Retsch GmbH, Germany) at 30 rotations/second for 1 min. Homogenised samples were stored individually in 15 mL amber polypropylene tubes (FALCON®, Labdirect from Thermoline Scientific, Australia) at − 20 °C for HPLC and NIRS analysis.

### HPLC reference methodology

The HPLC method used was an established routine analytical method from a Therapeutic Goods Administrated (TGA) accredited Analytical Research Laboratory (ARL) of Southern Cross University (Lismore, NSW, Australia).

Sample preparations and extractions were done in duplicate (technical replicate) by accurately weighing 500 mg of homogenised samples in 25 mL ethanol and agitating for 15 min in an ultrasound bath at 50/60 Hz at 150 W (Soniclean^®^, Australia). Samples were centrifuged at 3000 rpm for 10 min (Sigma 3-16L, Sigma Laborzentrifugen, GmbH, Osterode am Harz, Germany). The supernatant was collected into a 2 mL amber screw cap glass vial (Item #8010-0542, Agilent Technologies, Santa Clara, CA, USA) and stored at − 20 °C until analysis. A quality control (QC) sample containing known concentrations of 12 cannabinoids was prepared similarly and analysed with each batch as seen in the sample chromatograph in Supplementary Information Table S6.

A stock standard mix containing Δ9-THC, Δ9-THCA, CBD, and CBDA at a 0.1 mg/mL concentration was prepared in methanol. The stock solution was also diluted down to 0.01 mg/mL and 0.001 mg/mL in methanol. All three concentrations were injected using three different injection volumes: 1 µL, 2.5 µL and 5 µL to cover the following concentrations in 5 µL injection of the sample: 0.1, 0.05, 0.02, 0.01, 0.005, 0.002, 0.001, 0.0005 and 0.0002 mg/mL (a 9-point calibration curve prepared by plotting the peak area against the concentration). The test mix containing the unknown concentrations of additional cannabinoids (THCVA, CBDVA, CBGA, THCV, CBDV, CBG, CBN, and CBC) was also injected (5 µL) to confirm these cannabinoids’ identity using retention times. These additional cannabinoids were quantified using the calibration curves prepared for the four major cannabinoids. Chromatographic separation of cannabinoids was performed using an Agilent 1260 Infinity II high-performance-liquid chromatography (HPLC) (Agilent Technologies, Palo Alto, CA, USA). Separation was achieved using a Reverse Phase Phenomenex Luna, C18, 5 µ particle size, 250 mm × 4.6 mm column held at 40 °C. Equipped with a 1260 vacuum degasser, the HPLC system was 1260 quaternary pump, autoinjector and controlled using Agilent OpenLab Chemstation CDS software (version 2.5). Separated peaks were detected by a mass-spectrometer Agilent 6120 quadrupole mass detector (LC/MSDXT) and a diode array detector (Agilent UVDAD, 1260). The MSD was operated in a positive mode using the electrospray ionisation (ESI) source. Scan mass range was set at 100–500; fragmentor voltage at 135 V; capillary voltage at 3000 V; drying gas flow at 5.0 L/min (N_2_); vaporiser temperature at 200 °C; nebuliser pressure at 15 PSA; and drying gas temperature at 300 °C. Cannabinoids were quantified using the UVDAD at 210 nm and 274 nm, and the cannabinoids’ identity was confirmed using MSD.

Mobile phases used were: 0.005% (v/v) trifluoracetic acid (TFA) in MilliQ^®^ water (mobile phase A) and 0.005% (v/v) TFA in Acetonitrile (mobile phase B). The flowrate used was 0.6 mL/min. The column was equilibrated with 20% (v/v) A and 80% (v/v) B. The mobile phase’s composition was changed from 80% (v/v) B to 98% (v/v) B during the first 20 min of the gradient and maintained at 98% (v/v) B during the next three minutes. The composition was changed back from 98% (v/v) B to 80% (v/v) B during the next two minutes (25-min mark). The composition was maintained at 80% (v/v) B during the next three minutes to re-equilibrate the column.

### Data analysis and calculations

HPLC data analysis was performed using Agilent OpenLab Chemstation CDS processing software (version 2.5). Each cannabinoid in a chromatogram was identified based on their MS fragment, retention time and UVDAD spectra obtained from the reference solution. A processing method was prepared to integrate each peak based on retention time ± 0.1% and its UV response (210 nm for CBD, Δ9-THC and all other neutral cannabinoids, and 274 nm for CBDA, Δ9-THCA and all acid forms). Integrated peaks were exported to EXCEL (Microsoft 365, Washington, USA) for calculations. External standard calibrations using CBD, CBDA, Δ9-THC and Δ9-THCA standard mix were used to quantify 12 target cannabinoids in each sample. The calibration curve obtained for CBD at UVDAD 210 nm was used to calculate CBD, CBN, CBC, CBG and CBDV, as well as that for CBDA at UVDAD 274 nm, was used to calculate CBDA, CBDVA and CBGA. The calibration curve obtained for Δ9-THC at UVDAD 210 nm was used to calculate Δ9-THC and THCV, whereas the calibration curve obtained for Δ9-THCA at UVDAD 274 nm was used to calculate Δ9-THCA and THCVA). A 9-point calibration curve was plotted using concentration and peak area with an r^2^ acceptance above 0.999. The QC sample was used to estimate the cannabinoid’s validity for each sample batch. Cannabinoid concentrations for each sample were reported in % (w/w) and, concentration values obtained for each extract injected was checked to ensure their presence within the calibration range. Limit of detection (LOD) and limit of quantification (LOQ) for each cannabinoid were determined by the signal-to-noise ratio (ratio of 3 for LOD and 10 for LOQ). LOQ for all cannabinoids was estimated to be below 0.001% (w/w). See Supplementary Information Table S6 for cannabinoid retention times, wavelength, conversion factors, LOD and LOQ values. Cannabinoids below LOQ were reported as not-quantified for model building only.

### Spectroscopy workflow using reflectance near-infrared spectroscopy (NIRS)

A portable (260 × 80 × 80 mm) NIR diffuse reflectance spectrometer (HL-EVT9-Neo) was provided by Hone^®^ (Newcastle, NSW, Australia). The Hone Create platform supplied all model training and access to chemometric algorithms (version 1.0.1536). Metadata of all chemometric algorithms (pre and post- chemometric processing algorithms) used to create the predictive models for all 12 cannabinoids are listed in Supplementary Information Table S5.

The NIR produced a beam with a spot size of 10 mm-13 mm. Approximately 200 mg of sample (prepared as described in Sect. 3.2.2) was poured into a holding chamber and scanned in triplicate. Absorbance measurements were made in 1 nm increments between 1350 and 2500 nm. Scan settings used adaptive integration at 25,000 ms for lower bound parameters and 26,000 ms for upper bound parameters at ten iterations. Background integration was set to 2000 µs, whereas the NIR-Vis integration time was set at 1000 µs. During scanning, a background reading—to be subtracted from the signal produced by the holding chamber—was taken before scanning any samples and repeated every ten samples. The holding chamber and sensor were cleaned between samples using a KIM wipe (Kimtech®, Kimberly-Clark Worldwide INC) soaked in 100% ethanol. All results were recorded on a CSV file, including device settings, temperature, humidity and battery life.

### Chemometric model building

HPLC data on individual cannabinoids and corresponding *C. sativa* spectral data were used for model training. Samples were divided into two groups via randomization: (1) cross-validation (CV)—a training set (75%) designated for the iterative process of model training, and (2) hold-out (HV) validation—a validation set (25%) excluded from the model training. An initial assessment led to a cannabinoid limit value of 0.01% (w/w) except for CBDA and Δ9-THCA, which had a limit of 0.001% (w/w). Cannabinoids lower than these limits were removed from the model training after being considered detrimental to the model-building process. Several data transformations were undertaken iteratively to improve individual cannabinoid models, including unmerging technical scans (account for fluctuations and potential homogeneity issues), peak area normalisation, baseline shift corrections, derivatisation, smoothing and standard normal variate optimisation and obvious removal of outliers. Algorithms included random forest^63^, generalised linear model^64^, gradient boosting machine^65^ and deep neural networks^66^. These were used individually or as a stacked ensemble of all four.

## Supplementary Information

Supplementary Information 1.

Supplementary Information 2.

Supplementary Information 3.

## Data Availability

All data relevant to this study has been made available in the supplementary information, Tables S1 to S6.
