# Supplementary material for: Using a global diversity panel of Cannabis sativa L. to develop a near InfraRed-based chemometric application for cannabinoid quantification
Source: Sci Rep. 2023 Feb 8;13:2253. doi: 10.1038/s41598-023-29148-0 (PMC9908977; doi:10.1038/s41598-023-29148-0)
Supplement: Supplementary file 1 — Supplementary Information 1. [file 41598_2023_29148_MOESM1_ESM.docx]

Supplementary Information

Table S1 (.xls worksheet): Summary of the 249 samples used in this study from two germplasm collections, including accession passport data and cannabinoid contents.

Table S2 (.xls worksheet): Summary statistics of the cannabinoid content in % (w/w) for the full sample set quantified using HPLC.

Table S3 (.xls worksheet): Hierarchical clustering raw data.

Table S4 (.xls worksheet): Summary of the chemometric algorithms, model number and type for 12 individual cannabinoids.

Table S5 (.xls worksheet): Metadata of final models for 12 individual cannabinoids.

Table S6 (.xls worksheet): Summary of the HPLC quantification metrics.

Figure S1: Residual Plots for final models of all 12 target cannabinoids
