# Supplementary material for: Using a global diversity panel of Cannabis sativa L. to develop a near InfraRed-based chemometric application for cannabinoid quantification
Source: Sci Rep. 2023 Feb 8;13:2253. doi: 10.1038/s41598-023-29148-0 (PMC9908977; doi:10.1038/s41598-023-29148-0)
Supplement: Supplementary file 3 — Supplementary Information 3. [file 41598_2023_29148_MOESM3_ESM.pdf]

Supplementary Information Figure S1

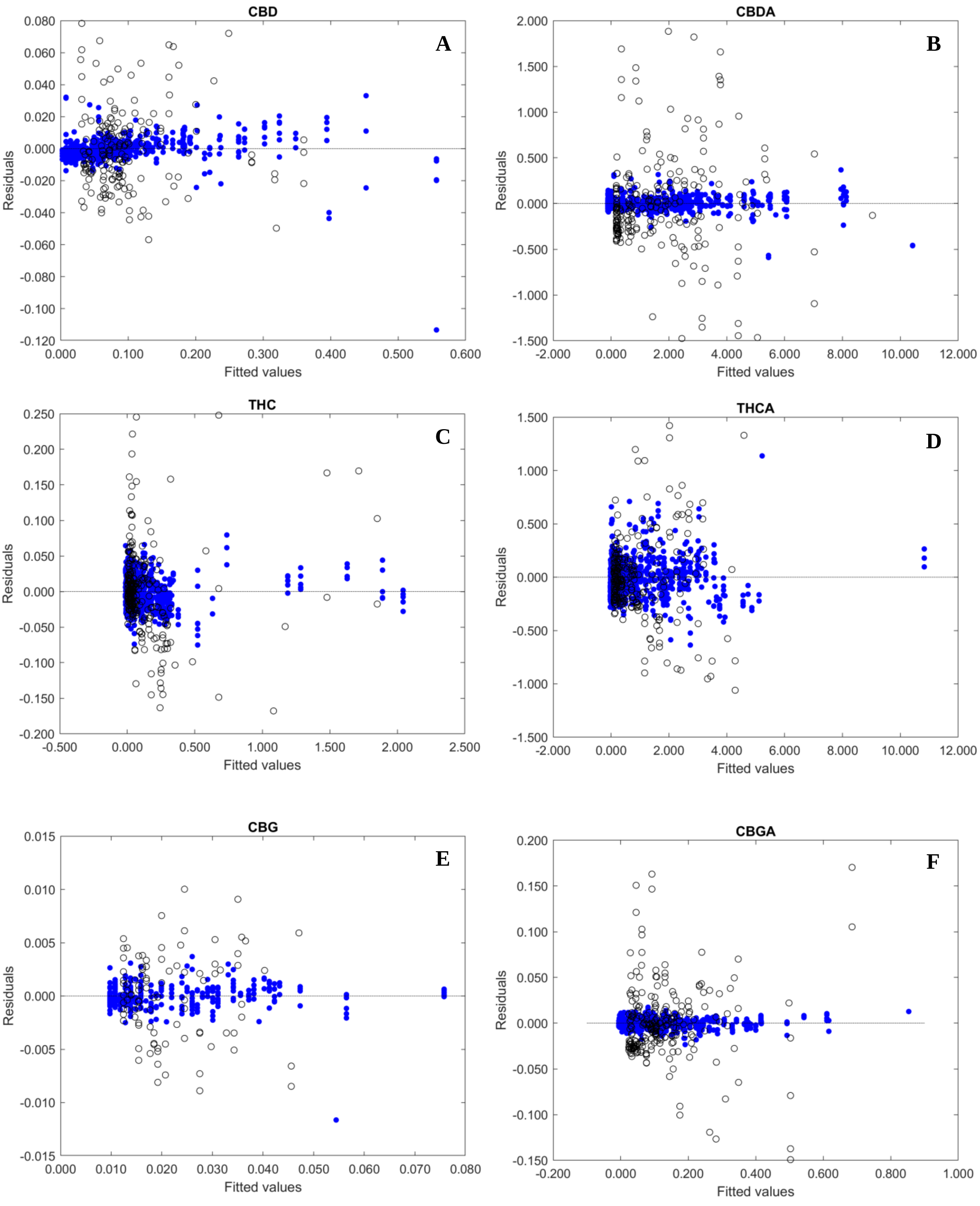

Supplementary Information Figure S1 (continued)

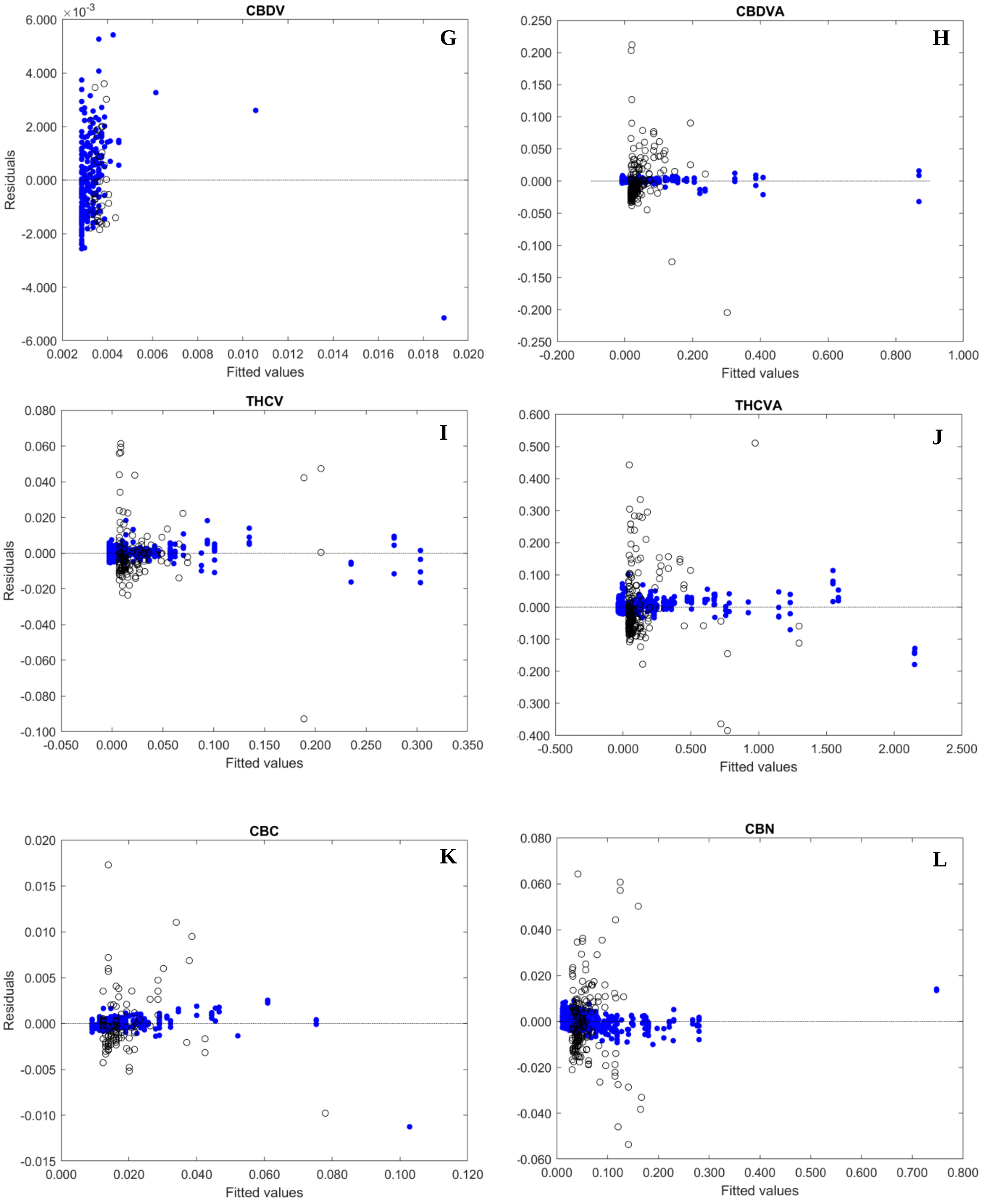

Figure S1: Residual plots for final models of all 12 target cannabinoids.
Residuals (y-axis) plotted against fitted values (x-axis) for Cross-validation (blue closed circles) and Hold-out validation (open circles) data of the 12 target Cannabinoids: A.) Cannabidiol (CBD) B.) Cannabidiolic acid (CBDA), C.) Δ9-tetrahydrocannabinol (Δ9-THC), D.) Δ9-tetrahydrocannabinolic acid (Δ9-THCA), E.) Cannabigerol (CBG), F.) Cannabigerolic acid (CBGA), G.) Cannabidivarin (CBDV), H.) Cannabidivarinic acid (CBDVA), I.) Tetrahydrocannabivarin (THCV), J.) Tetrahydrocannabivarinic acid (THCVA), K.) Cannabichromene (CBC), L.) Cannabinol (CBN).
